# Brownian dynamics simulation of analytical ultracentrifugation experiments

**DOI:** 10.1186/2046-1682-4-6

**Published:** 2011-03-02

**Authors:** AI Díez, A Ortega, J Garcia de la Tore

**Affiliations:** 1 Departamento de Química Física, Facultad de Química, Universidad de Murcia, 30071 Murcia, Spain

## Abstract

**Background:**

We have devised a protocol for the Brownian dynamics simulation of an analytical ultracentrifugation experiment that allows for an accurate and efficient prediction of the time-dependent concentration profiles, *c*(*r, t*) in the ultracentrifuge cell. The procedure accounts for the back-diffusion, described as a Brownian motion that superimposes to the centrifugal drift, and considers the sector-shaped geometry of the cell and the boundaries imposed by the meniscus and bottom.

**Results:**

Simulations are carried out for four molecules covering a wide range of the ratio of sedimentation and diffusion coefficients. The evaluation is done by extracting the molecular parameters that were initially employed in the simulation by analyzing the profiles with an independent tool, the well-proved SEDFIT software. The code of simulation algorithm has been parallelized in order to take advantage of current multi-core computers.

**Conclusions:**

Our Brownian dynamics simulation procedure may be considered as an alternative to other predictors based in numerical solutions of the Lamm equation, and its efficiency could make it useful in the most relevant, inverse problem, which is that of extracting the molecular parameters from experimentally determined concentration profiles.

## Background

Since the invention of the analytical ultracentrifuge by Svedberg [1], the technique of analytical ultracentrifugation (AUC) has been a classical - and, thanks to advances in instrumentation and analysis software, it is still a most modern - technique for characterization of macromolecules and nanoparticles in solution. The reader may grasp the recent importance of this field in monographs [2-4] and thematic issues of other journals [5-7].

In the AUC, particles move under influence of a centrifugal field, caused by rotation of the sample with angular velocity *ω*, which produces a centrifugal force (corrected by buoyancy) equal to $\omega^{2}rm(1-\bar{v}\rho )$, where *r *is the instantaneous distance from the particle to the rotation axis, *m *is the mass of the particle (*m *= *M*/*N_A _*where *M *is the molecular weight and *N_A _*is Avogadro's number), $\bar{v}$ is the partial specific volume of the solute particles and *ρ *is the solution density (nearly equal to the solvent density, if the solution is dilute). The velocity that the solute particles may acquire due to this effect is proportional to the centrifugal acceleration, *υ *= *sω*^2^*r*, where the *s *is the sedimentation coefficient, and modulated also by the friction coefficient *f *of the particle in the viscous solvent:

(1) $$s=\frac{v}{\omega^{2}r}=\frac{m(1-\bar{v}\rho )}{f}=\frac{M(1-\bar{v}\rho )}{N_{A}f}$$

If this were the only action on the solute particles, their motion would be purely deterministic. If *r*(*t*) is the radial position of the particle at time *t*, one easily finds (considering that *υ *= *dr*/*dt*) that the position after some time, Δ*t*, would be given by

(2) $$\text{ln}\frac{r(t+\Delta t)}{r(t)}=s\omega^{2}\Delta t$$

or

(3) $$r(t+\Delta t)=r(t)\exp(s\omega^{2}\Delta t)$$

Even if the initial (loading) concentration in the AUC cell is uniform, i.e., constant from the meniscus to the bottom in the AUC cell, which are placed at distances *r_m _*and *r_b_*, respectively, from the rotor, centrifugation will provoke some transport of the solute particles, and therefore a concentration gradient will be produced. This gradient will, in turn, generate a counterflow of solute in the direction of decreasing concentration, i.e., contrary to the centrifugal velocity. Macroscopically, at a point *r *the counterflow would be determined by the first law of Fick, *J *= −*D*∇*c*(*r, t*), where *D *is the diffusion coefficient, related to both *f *and *s *by the Einstein and Svedberg equations, respectively:

(4) $$D=\frac{k_{B}T}{f}$$

(5) $$\frac{s}{D}=\frac{M(1-\bar{v}\rho )}{RT}$$

where *k_B _*is Boltzmann constant, *T *is the absolute temperature and *R *= *k_B_N_A _*is the constant of perfect gases.

The AUC experiment yields the concentration profile as a function of time, i.e., the two-variables function *c*(*r, t*). It is from the analysis of this function that the information of interest about the solute particles, i.e., the values of *s*, *D *and *M *can be determined. When the diffusional counterflow can be ignored - e.g., when the sedimentation is carried out at extremely large *ω*, or for massive particles having a great *s*/*D *ratio - such analysis is simply based on eq. 2, as described in textbooks [8], and provides a value of *s*. However, in general cases, particularly when the diffusional effect is influential, and therefore one could determine not only *s*, but also *D*, and *M *therefrom, a rigorous consideration of both effects is required. The time-dependent concentration profile *c*(*r, t*) is determined by the balance between the centrifugal and diffusional effects. Classically, this has been expressed in macroscopic terms, in the form of the so-called Lamm equation [9], which expressed the balance between the centrifugal drift and the backwards flux that, according to the Fick law, has to occur because of the generated concentration gradient. The Lamm equation reads:

(6) $$\frac{\partial c}{\partial t}=\frac{1}{r}\frac{\partial }{\partial r}\left[rD\frac{\partial c}{\partial r}-s\omega^{2}r^{2}c\right]$$

Eq. 6 is written in cylindrical coordinates, because the AUC geometry is radial, and sector-shaped cells are used (devised so that the radial trajectories would not collide with the lateral walls).

In spite of the basic nature of the concepts involved, the solution of the Lamm partial differential equation, with the geometry and boundary conditions of the AUC experiment is extremely difficult, and requires numerical methods [10,11] based on time discretization and a finite-elements description of the AUC cell. [10]. Nonetheless, in modern AUC analysis programs, like SEDFIT [12], a Lamm-equation solver is embodied, enabling the prediction of computed *c*(*r, t*) for estimations of *s*, *D *and *M*, which are to be optimized as to fit the experimental *c*(*r, t*). In these procedures (apart from the fitting or optimization algorithms, a central piece is the prediction of *c*(*r, t*).

In this paper we investigate an alternative predictor of AUC concentration profiles. Instead of starting from the balance of the macroscopic flows established by the Lamm equation, we consider a microscopic description of the motion of particles under the simultaneous effect of a deterministic force, and the random forces characteristic of Brownian motion, so that the latter replaces the Fick's law description of macroscopic diffusion. In a simple (and somewhat naïve) approach, we formulate a Brownian dynamics algorithm to simulate the trajectories of particles. With our microscopic perspective, our method also discretizes time (Brownian simulation steps) and instead of finite elements use discrete particles. Carrying out such simulation for a sufficiently large number of particles, we can determine the time-dependent concentration profile in the AUC cell. In the next section we describe the procedure and demonstrate adequacy of its results, and finally we shall discuss on its performance, eventual advantages and possible extensions for further applications.

## Methods

### The simulated system

**Figure 1:**
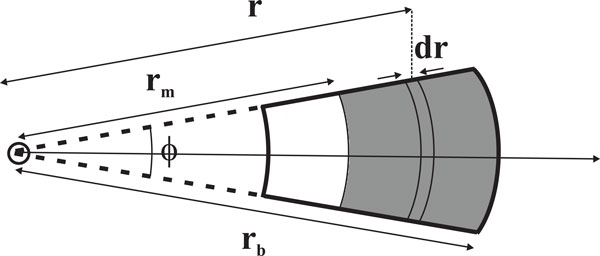
**AUC cell scheme**. Scheme of AUC experiment with a typical sector-shaped cell, showing the position of the meniscus *r_m_*, botton *r_b _*and the instantaneous position of a solute particle *r*.

We consider a solution of concentration *c*_0_, initially uniform at *t *= 0, in a sector-shaped cell (Figure 1). In the simulation, the solute is represented by a collection of a large number of particles *N_part_*. The radial distance is discretized in *N_r _*intervals of width *χ *= (*r_b _*− *r_m_*)/*N_r_*. Thus the cell is divided in slices of width *χ *and transversal area *ϕh*, where *ϕ *is the angle of the sector comprising the cell, and *h *its height, so that the volume of a slice placed at distance *r_i_*, *i *= 1, ... *N_r_*, is *ϕhχr_i_*. The concentration profile is to be calculated during a total time *T*, at a series of *N_t _*time intervals, of duration *τ*, so that *T *= *N_t_τ *and *t_j _*= *jτ*, *j *= 1, ... *N_t_*.

The mass concentration *c*(*r_i_*, *t_j_*) in the real system is proportional to the number concentration in the simulated system:

(7) $$c(r_{i},t_{j})=Q\frac{n(i,j)}{\phi h\chi r_{i}}$$

where *Q *is a proportionality constant which can be fixed imposing the condition for the initial uniform concentration over the whole volume of the cell, $\frac{1}{2}\phi h(r_{b}^{2}-r_{m}^{2})$:

(8) $$c_{0}=Q\frac{2N_{part}}{\phi h(r_{b}^{2}-r_{m}^{2})}$$

so that combining eq. 7 with 8 we readily find,

(9) $$\frac{c(r_{i},t_{j})}{c_{0}}=\frac{\left(r_{b}^{2}-r_{m}^{2}\right)}{2\chi }\frac{n(i,j)/N_{part}}{r_{i}}$$

Note that, apart from several constants relative to the instrument or the simulation the concentration is not determined by *n*(*i, j*), but by *n*(*i, j*)/*r_i_*. This takes into account what is called in the AUC terminology, the radial dilution effect.

What we simulate corresponds to a highly diluted solution, in which there are no particle interactions. Thus we can generate trajectories of individual particles, independent of each other.

### Brownian dynamics simulation

The main aspect of the Brownian dynamics (BD) simulation is the propagation of the particle trajectories. As we are considering non-interacting particles, the trajectory of one particle is independent of that any other, so that those quantities are the diffusion coefficient, *D*, and the force *F*, acting on the particle. In the present problem, we could begin with a basic algorithmic procedure in which the time step, *δt *is rather small, so that neither of the quantities determining the step change appreciably during *δt*. Then, the running algorithm for the particle's position would be:

(10) $$r(t+\delta t)=r(t)+\delta r_{sed}+\delta r_{Brow}$$

where *δr_sed _*= *ω*^2^*srδt *is the deterministic sedimentation drift of the particle with instantaneous, position-dependent velocity *ω*^2^*sr*, while the random Brownian displacement has zero mean and variance $<\delta r_{Brow}^{2}>=2D\delta t$.

Although - as it will be verified later on - the end effects, at the solution meniscus and the bottom of the cell are of minor importance we take them into account. As the description sedimentation and Brownian motion near boundaries or walls seems problematic, we adopt *ad hoc *criteria. As for the meniscus, if after the step *r *<*r_m_*, we set *r *= *r_m_*. Regarding the bottom, if *r *>*r_b_*, the particle had hit the bottom of the cell during the step; then we assume it should bounce and correct the position, taking *r *− 2(*r *− *r_b_*) = 2*r_b _*− *r*. After testing that this algorithm, in which the trajectory is divided in a very large number of small time steps, predicts correctly the concentration profiles (see below) we intended to devise a procedure with larger times steps, which would be computationally faster. The displacement over a large time step Δ*t *is the result of the integration of the small increments in eq. 10, so we can write

(11) $$r(t+\Delta t)=r(t)+\Delta r_{sed}+\Delta r_{Brow}$$

During the large step the sedimentation velocity changes as *r *changes, but this change is deterministic, and as mentioned above the sedimentation drift is easily integrated as indicated in eq. 12

(12) $$\Delta r_{sed}=r(t)[1-\exp(s\omega^{2}\Delta t)]$$

while, thanks to the fractal nature of the Brownian motion, the Brownian step follows the same law over the long time, Δ*r_Brow _*being a random number of zero mean and variance

(13) $$<\Delta r_{Brow}^{2}>=2D\Delta t$$

Thus the algorithm based on eqs. 11, 12 and 13 could be applicable to arbitrarily large time steps (even as large as the time interval *τ *between registers). This is essentially true if there were no end effects, i.e., in infinite, unbound AUC cell. For the sake of simplicity, we still adopt the simple criteria that particles stop at the meniscus and bounce at the bottom. Thus the only defect introduced by this procedure would be an inaccurate prediction of the concentration near the meniscus and bottom. In this regard, we note that the end-effects also affect other prediction procedures, like those based in Lamm-equation solvers, and influence the experiment itself, so that it is a common practice to discard the two terminal regions in the analysis of AUC experiments.

### Procedure

Summarizing from the previous description, Brownian dynamics trajectories are simulated for a large number of particles, *N_part_*. The trajectory of one particle is monitored, determining at successive times *t_j _*the interval of radial position *r_j_*. Then the counter for those interval and position is increased *n*(*i, j*) → n(*i, j*) + 1.

The initial position of the particle is assigned according to the uniform concentration in the sector-shaped cell. As the number of particles in a slice of thickness *χ *is proportional to r, the probability of having (in the uniform solution) a particle at a distance *r *is *p*(*r*) ∝ *r*. Integrating from *r_m _*to *r_b _*gives

(14) $$p(r)=\frac{2}{r_{b}^{2}-r_{m}^{2}}r$$

The initial position should be a random number obeying to the probability density expressed by eq. 14, and accumulative distribution function

(15) $$F(r)=\int_{r_{m}}^{r}p(r)dr=\frac{r^{2}-r_{m}^{2}}{r_{b}^{2}-r_{m}^{2}}$$

Thus we generate a random number with uniform distribution *u *∈ (0, 1) which is equated to *F *(*r*) to obtain the initial position.

(16) $$r=\sqrt{u(r_{b}^{2}\;-\;r_{m}^{2})\;+\;r_{m}^{2}}$$

If *t_run _*is the total time of the experiment (usually, several hours), the simulation for each particle consists of a number of steps *N_steps _*= *t_run_*/Δ*t*. For recording purposes, a number of registers (scans in the AUC experiment), equal to *N_scans_T *= *t_run_*/*τ *is made, observing the radial distance, and therefrom the index *i*, of the slice at which the particle is at that instant, *j *= *t_run_*/*τ*. Then one unit is added to the counter, *n*(*i*, *j*). Trajectories are simulated for a sufficiently large number of particles *N_part_*. At the end of the simulations, concentration profiles *c*(*r_i_, t_j_*) are obtained from the *n*(*i, j*) counter using eq. 9.

### Simulation data

The data employed in the simulation reflect those of real AUC experiments. The geometry of the cell is given by *r_m _*= 5.80 cm, *r_b _*= 7.20 cm and sector angle *ϕ *= 3 degrees (0.05 radians). The rotor speed *ω *and the duration of the experiment *t_run _*was varied depending on the molecule being simulated, and the mode (velocity or equilibrium) of the experiment. In some cases also the meniscus position was varied for equilibrium experiments. We found that *N_part _*= 10^5 ^particles suffices to obtain a rather low level of noise as it will be shown below.

Simulations were done for four different molecules covering an extremely large range of sedimentation to diffusion coefficients (i.e., in a very wide range of molecular mass), so that the extreme cases of vanishing and intense diffusion broadening are considered in our study. This includes a quite small molecule - *γ*-cyclodextrin - a small globular protein - lysozyme - a moderately long flexible polymer - poly(ethylene oxide) - and a very long and stiff DNA from T7 bacteriophage. Values for *s*, *D *and *M *are taken from the literature [8,13-16], and listed in Tables 1 and 2, along with the conditions regarding spinning time and velocity for each sample in both modes, for velocity and equilibrium sedimentation experiments

**Table 1:** Experimental properties

Molecule	*γ*-cyclodextrin	Lysozyme	Polyoxyethylene	DNA T7
$1-\bar{v}\rho$	0.333	0.298	0.170	0.450
10^−3^*M *(g/mol)	1.50	14.3	300	26 000
*s *(S)	0.53	1.89	2.30	31.8
10^7^*D *(cm^3^/g)	27.0	10.8	1.10	0.066
*ω *(rpm)	55 000	40 000	40 000	10 000
*t_run _*(secs.)	40 000	40 000	40 000	60 000
10^−3^*M_calc_*(g/mol)	1.44 (-4)	13.9 (-3)	300 (0)	23 278 (-10)
*s_calc _*(S)	0.54 (+2)	1.84 (-3)	2.26 (-2)	31.4 (-1)
10^7^*D_calc _*(cm^3^/g)	27.4 (+1)	10.8 (0)	1.08 (-2)	0.073 (+11)

Molecules and the experimental properties used as input in the simulation of sedimentation velocity experiments, and properties recovered from the simulation profiles after the SEDFIT analysis. Numbers between parenthesis are the percent deviations of the recovered values from those used as input for the simulations.

**Table 2:** Equilibrium experiments

Molecule	*γ*-cyclodextrin	Lysozyme	Polyoxyethylene
*ω *(rpm)	30 000	15 000	5 000
*t_run _*(secs.)	300 000	500 000	500 000
10^−3^*M_calc_*(g/mol)	1.27 (-16)	12.6(-12)	250.0 (-17)

Conditions for simulation of sedimentation equilibrium experiments, and molecular weight recovered from the analysis of the sedimentation profiles using eq. 17. Numbers between parenthesis are as in Table 1.

## Results and discussion

**Figure 2:**
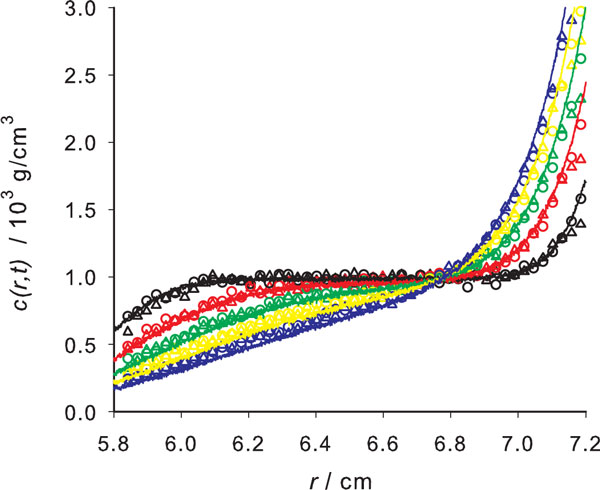
**A sedimentation velocity simulation**. Concentration profiles for sedimentation velocity of *γ*-cyclodextrin (data in Table 1), at times 4 444 s (black), 13 333 s (red), 22 222 s (green), 31 111 s (yellow) and 40 000 s (blue), showing simulation results with *N_steps _*= 4000 (open circles) and *N_steps _*= 50 (open triangles) along with SEDFIT predicted profiles (thin lines).

First of all, we checked that the results from the algorithm with a small number of large time steps, eq. 11 give practically the same results than that with many small steps, eq. 10, thus confirming the efficiency of the former. This was evident in all cases simulated, as shown for example in Figure 2.

In order to evaluate the accuracy of the results for the prediction of sedimentation profiles in the velocity mode, we have compared them with calculations with the well-known SEDFIT software [12]. This tool, which includes a sophisticated Lamm-equation solver has an utility for predicting the concentration profiles. Comparison of our simulated profiles with the SEDFIT predictions resulted in a good agreement, as shown in Figure 2. The determination of concentration profiles has the final purpose of determining the molecular parameters of interest, so the most relevant evaluation of their prediction is the confirmation of whether their analysis provides correct values for those parameters. In order to do so with our simulation results, we employed the analysis tool of SEDFIT as an independent and robust criterion. Indeed, SEDFIT uses a reliable Lamm-equation solver to determine the parameters by means of powerful optimization algorithms. In the single-species mode of SEDFIT, the program provides the values of the molecular weight, *M*, and the sedimentation and diffusion coefficients, *s *and *D *of the sample. We made the SEDFIT analysis with typically 50 scans (*t *values) each covering 1400 radial positions *r *values.

In Table 1 we report the *s*, *M*, *D *and values for the four samples considered in our study. We note the high accuracy of the recovered values of these three parameters, reflected in their very small deviations (particularly in the case of *s*) from the values employed as input in the simulation. The agreement is good for the four cases, which, as indicated above, cover a wide range of the sedimentation-to-diffusion ratio and molecular mass, including in the velocity experiments a molecule as small as cyclodextrin.

**Figure 3:**
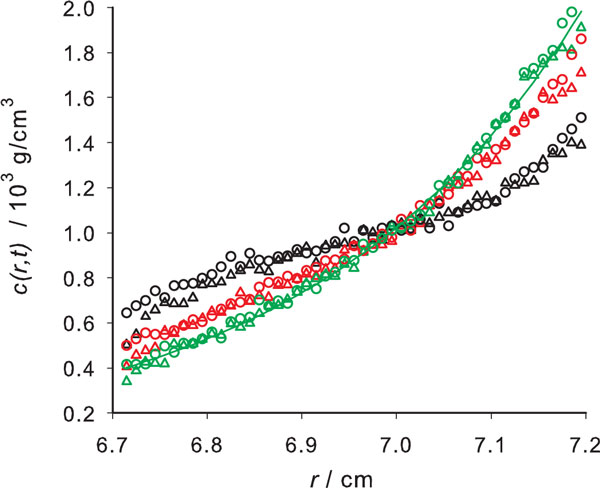
**A sedimentation equilibrium simulation**. Concentration profiles for sedimentation equilibrium of PEO (data in Table 1), at times 111 111 s (black), 277 778 s (red) and 500 000 s (green), showing simulation results with *N_steps _*= 4000 (open circles) and *N_steps _*= 50 (open triangles). The thin lines indicate the profiles obtained applying the classical sedimentation equation 17.

The simulated runs in the equilibrium mode were conducted for long times, following the evolution of the simulation profile to make sure that a further extension of the run time would not change the resulting final equilibrium *c*(*r*) profile. An example is presented in Figure 3. For the analysis of the simulated runs in the equilibrium mode, we adopted the classical equation [8]:

(17) $$c(r)=c_{0}\exp\left[\frac{\omega^{2}M(1-\bar{v}\rho )(r^{2}-r_{m}^{2})}{2RT}\right]$$

which, as usual, is linearized in the form of a plot of ln *c*(*r*) vs. $r^{2}-r_{m}^{2}$ whose slope is $\omega^{2}M(1-\bar{v}\rho )/(2RT)$, from which the molecular weight *M *is extracted. The results are listed in Table 2. Here we observe that the recovered values of *M *are not as good as those obtained in the velocity mode, with deviations of about 15%. Among other reasons (like the smaller amount of information resulting from equilibrium experiments, and the errors inherent to the determination of the slope in the fit to the linearized equation) this may be because the simple eq. 17 neglects radial dilution.

## Computing details

The extreme simplicity of the algorithm that simulates the trajectory of one molecule makes the simulation scheme very well adapted for parallelization, thus taking benefit of present multi-core platforms, because each trajectory can be generated in a separate thread/core. We have implemented OpenMP directives in our Fortran 90 code and tested the performance in a DELL T5500 workstation with two Intel Xeon X5660 processors. 1000 simultaneous simulation with *N_part _*= 10^5^, *N_r _*= 100 radial positions and *N_t _*= 50 recorded scans took 46.5 CPU seconds. Broadly speaking, our algorithm, which is easily parallelized, is able to run one thousand *c*(*r, t*) calculations in less than one minute. The computing speed of the algorithm is crucial in its main use, namely, in the analysis of *c*(*r, t*) experimental profiles by any kind of fitting to computed profiles, and the speed of our procedure seems suitable for that purpose.

A nice feature of the Brownian simulation of ultracentrifugation is that it allows to visualize the simulated trajectories using computer graphics. This may be of utility for demonstrative purposes (e.g. in teaching AUC principles). We have produced two videos, showing the evolution of the solute particles in the AUC as the sedimentation proceeds, one at high rotor speed, in the velocity mode, and another at low speed, that reaches the sedimentation-diffusion equilibrium. These videos accompany this paper as additional files 1 and 2.

## Conclusions

In this work we have presented a simple procedure, based on a Brownian dynamics, microscopic simulation, for predicting the time/position-dependence of concentration (concentration profiles *c*(*r, t*)) during the AUC experiment, which can be regarded as an alternative to the numerical solution of the macroscopic Lamm equation. The correctness of the procedure has been tested comparing the profiles with those computed by the Lamm-equation solution, and the concordance of the molecular parameters recovered from them with those used in the simulation.

Having presented this proof-of-concept, some advantages of our scheme can be hinted - although they all remain to be evaluated in future work. An important aspect to be considered is computational efficiency. As commented above, our computational procedure has the feature of being perfectly parallelizable. A comparison with the numerical solution of Lamm solution requires further the labor, implementing codes for those procedures and making a side-to-side analysis of computing speed and requirements. Further work is planned in this regard.

Apart from computing efficiency, the BD scheme has the potential advantage that it can treat easily cases of arbitrary complexity. In our proof-of-concept we have restricted ourselves to the simplest case of identical, non interacting particles (extension to different but still non-interacting particles is trivial). An extraordinary utility of AUC is the characterization of macromolecular interactions. For problems with interacting particles, the BD scheme can be easily adapted. In BD, detailed interactions between the particles can be modeled whereas the continuum method only allows for averaged density dependent potentials. Even if computing efficiency would suffer in such, more complex problems, still the BD approach, which is based on first principles and allows explicit description of interaction between particles (or the effect of special conditions in the AUC experiments) may be a valuable tool for testing other approaches.

## Computer programs

Fortran 90 source code with OpenMP directives will be freely downloaded from our web site, http://leonardo.inf.um.es/macromol/

## Authors' contributions

AID was involved in the implementation, programming and calculations. AO was involved in the programming and manuscript writing, correcting and editing. JGT conceived the project and the study hypothesis, designed the program and was involved in the manuscript writing. All authors read and approved the final manuscript.

## Supplementary Material

Additional file 1 **Sedimentation velocity experiment of lysozyme**: Movie showing the trajectory followed by 1000 molecules of lysozyme, represented as green spheres in a typical ultracentrifuge cell, *r_m _*= 5.8 cm, *r_b _*= 7.2 cm in a sedimentation velocity experiment with *ω *= 40000 rpm and 54 000 s, at 20°C. The white spheres represent the water solvent. Click here for file

Additional file 2 **Sedimentation equilibrium experiment of cyclodextrin**: Movie showing the trajectory followed by 1000 molecules of cyclodextrin, represented as red spheres in a typical ultracentrifuge cell, *r_m _*= 5.8 cm, *r_b _*= 7.2 cm in a sedimentation equilibrium experiment with *ω *= 30000 rpm and 300 000 s, at 20°C. The white spheres represent the water solvent. Click here for file
